# New treatment options for metastatic renal cell carcinoma with prior anti-angiogenesis therapy

**DOI:** 10.1186/s13045-016-0374-y

**Published:** 2017-02-02

**Authors:** Kevin Zarrabi, Chunhui Fang, Shenhong Wu

**Affiliations:** 1 Department of Medicine, Stony Brook University Hospital, 9447 Suny, Stony Brook, NY 11794-9447 USA; 2 Division of Hematology/Oncology, Department of Medicine, Northport VA Medical Center, Northport, NY USA

**Keywords:** Renal cell carcinoma, Anti-angiogenesis, Lenvatinib, Cabozantinib, Nivolumab

## Abstract

Angiogenesis is a critical process in the progression of advanced renal cell carcinoma. Agents targeting angiogenesis have played a primary role in the treatment of metastatic renal cell carcinoma. However, resistance to anti-angiogenesis therapy almost always occurs, and major progress has been made in understanding its underlying molecular mechanism. Axitinib and everolimus have been used extensively in patients whom have had disease progression after prior anti-angiogenesis therapy. Recently, several new agents have been shown to improve overall survival in comparison with everolimus. This review provides an in-depth summary of drugs employable in the clinical setting, the rationale to their use, and the studies conducted leading to their approval for use and provides perspective on the paradigm shift in the treatment of renal cell carcinoma. Highlighted are the newly approved agents cabozantinib, nivolumab, and lenvatinib for advanced renal cell carcinoma patients treated with prior anti-angiogenesis therapy.

## Background

Each year, over 320,000 individuals will be diagnosed with renal cell carcinoma (RCC) accounting for an annual death toll of over 140,000 people. The incidence of RCC has steadily risen over the past 10 years and accounts for 2–3% of all adult malignancies [1]. Upon the diagnosis of RCC, curative surgery is an option for those with early stage localized tumors. However, localized disease may undergo early hematogenous dissemination leading to metastasis. Sites of early metastases include the lungs, lymph nodes, liver, bone, and brain. RCC can also metastasize to the adrenal glands, the contralateral kidney, although less commonly [2]. Individuals with advanced disease face high rates of morbidity and mortality with a median 5-year survival rate of 53% for stage III disease and 8% for metastatic disease [3, 4]. However, death rates from RCC have remained stable or decreased in most countries with advanced healthcare [5]. This steady decline can be attributed to the advent of new biological agents rapidly developed over the past few years that are readily employable in the clinical setting.

RCC is a heterogeneous disease and is subcategorized based on histological and cytogenetic signatures, with 80% characterized as clear cell renal cell carcinoma (ccRCC) and 20% as non-clear cell carcinoma (nccRCC) [6]. Both types can occur either sporadically or due to a hereditary predisposition. However, both forms are associated with gene mutations to the short arm of chromosome 3 and specifically to the *VHL* tumor suppressor gene [7, 8]. In the natural setting, the *VHL* gene encodes the substrate recognition module of a ubiquitin ligase that targets hypoxia-inducible factor (HIF) for destruction in the presence of oxygen. However, *VHL* is mutated or methylated in up to 90% of patients with ccRCC [9]. When the *VHL* gene is mutated, its tumor suppressor function is lost and HIF accumulates to high levels, leading to the activation of multiple genes including vascular endothelial growth factor (VEGF) and platelet-derived growth factor (PDGF). Ultimately, this cascade of events culminates in unregulated cell growth, uncontrolled angiogenesis, and increased tumor-cell invasion.

Elucidation of this underlying pathway has led to the development of a number of target-based therapies for patients with advanced RCC. Prior to the advances in therapeutics seen over the last decade, the mainstay of treatment for metastatic disease was cytokine-based treatment with high dose interleukin-2 (IL-2) and interferon-alpha (IFN-α) after their FDA approval in the 1990s [10]. Although this therapy regimen produced objective responses, there were significant toxicities, treatment benefit was only seen in 5–15% of patients, and outcome for the majority of patients was poor [11, 12]. Since 2004, the advances in target-based therapy and immunotherapy modalities have created a paradigm shift in the treatment of RCC. These agents have had a remarkable effect on patient outcomes with increased progression-free survival rates; however, virtually all patients eventually develop the progression of disease [7]. The high likelihood of disease progression remains a challenge due to therapeutic resistance. Refractory disease is currently being managed with sequentially changing therapy, but morbidity and mortality remain high. Herein, we review the most up-to-date practices and emerging therapies for the treatment of refractory RCC after anti-angiogenesis therapy and focus on newly approved agents including cabozantinib, nivolumab, and lenvatinib.

## The primary role of anti-angiogenesis in first-line therapy for mRCC

The armamentarium of agents approved for the first-line treatment of metastatic RCC (mRCC) has rapidly developed over the years and now includes the small-molecule VEGF tyrosine kinase inhibitor (TKI)-sunitinib and pazopanib, a monoclonal antibody targeting VEGF-bevacizumab in combination with interferon, and an mammalian target of rapamycin (mTOR) inhibitor-temsirolimus, as well as high dose IL-2. In the recent past, the approach to the treatment of patients with mRCC entailed sequential employment of agents targeting VEGF or mTOR pathways. Agents with anti-angiogenesis properties have become the mainstay of initial therapy for advanced RCC due to their preferable efficacy and toxicity profile. The current level 1 recommendation from the National Comprehensive Cancer Network (NCCN) and the European Association or Urology is the use of oral, multi-target, tyrosine kinase inhibitors (TKIs)—specifically sunitinib and pazopanib—in the first-line setting [13, 14].

### VEGF-targeted tyrosine kinase inhibitors

Sunitinib is an orally administered multi-target TKI of VEGFR, PDGFR, and c-Kit and is generally well tolerated. Originally, sunitinib demonstrated a progression-free survival (PFS) of 8.3 months in patients who progressed on one line of cytokine-based therapy. This led to a follow-up study on its use as a first-line agent [15]. A pivotal phase III clinical trial involving 750 treatment-naïve patients was conducted to compare sunitinib to IFN-α as first-line treatment for mRCC. The study met its primary endpoint, PFS, and sunitinib demonstrated a superior PFS of 11 months compared to 5 months with IFN-α. Sunitinib also proved superior in overall survival (OS) with 26.4 months as compared to 21.8 months for IFN-α [15]. The side-effect profile has been studied thoroughly, and common adverse effects include hypertension (12%), fatigue (11%), diarrhea (9%), and hand-foot syndrome (9%) [16].

In light of its favorable safety and tolerability profile, a second TKI, pazopanib, was studied in the mRCC population [17]. A phase III, double-blind, placebo-controlled study was designed to evaluate pazopanib in 435 treatment-naïve or cytokine-pretreated patients. Pazopanib was shown to prolong mPFS compared to placebo (11.1 vs. 2.8 months) and to cytokine-pretreatment (7.4 vs. 4.2 months). Sternberg et al. reported a median OS of 22.9 months with pazopanib and 20.5 months with placebo [18]. Common pazopanib toxicities during this trial included diarrhea (52%), hypertension (40%), hair color changes (38%), and nausea (26%) [17].

In order to select the optimal first-line therapy, head-to-head comparison studies were performed for these two TKIs. In 2013, Motzer et al. presented the COMPARZ trial, a global, phase III, randomized, open-label trial comparing sunitinib and pazopanib as first-line agents. Both agents performed similarly, and the mPFS of pazopanib was 10.5 months compared to 10.2 months for sunitinib. OS analysis resulted in 28.4 and 29.3 months for pazopanib and sunitinib, respectively. Overall clinical efficacy was not discernable between the two agents, and pazopanib was concluded to be non-inferior to sunitinib [19]. In addition to the COMPARZ trial, the PISCES study investigated patient-reported outcomes with regard to pazopanib and sunitinib tolerability and patient preference. Stratification of data from this questionnaire-based study revealed a significant number of patients preferred pazopanib over sunitinib. There was a 49% reported difference in preference between the two agents [20]. Interestingly, both agents have uniquely different side-effect profiles despite similar target pathways and mechanisms of action. Pazopanib has a lower incidence of hand-foot syndrome, fatigue, and myelosupression but notable for more frequent hepatic injury. Such a discernable difference in toxicities has been used as a tool to tailor therapy for mRCC patients based on their respective comorbidities [21].

Other TKIs include sorafenib, axitinib, and cabozantinib which were also tested in the first-line setting. Sorafenib was introduced as the first targeted therapy for mRCC in 2004 [22]. An oral, multi-kinase inhibitor of tumor-cell proliferation and tumor angiogenesis, sorafenib, is currently indicated for the treatment of renal, liver, and thyroid cancers. A phase III study, presented as the TARGET trial, enlisted 903 mRCC patients with disease refractory to high dose IL-2 and IFN-α and stratified patients to a placebo or sorafenib treatment group. Sorafenib outperformed placebo in mPFS 5.5 to 2.8 months, respectively [23]. Originally, no significance was found in OS from the study data; however, further analysis censoring placebo patients with crossover demonstrated a survival advantage for sorafenib-treated patients. Moreover, the TARGET trial also provided data suggesting VEGF levels are prognostic for mPFS and OS in mRCC [24]. It must be noted, however, that sorafenib did not improve PFS when compared to IFN-α-2a in the first-line setting [25].

Axitinib, initially approved in the second-line setting, was also studied as a first-line agent in comparison with sorafenib in a randomized, open-label, phase III clinical trial. Although axitinib demonstrated clinical activity with an acceptable safety profile, there was no significant difference in mPFS in patients with treatment-naïve mRCC compared with those treated with sorafenib in the first-line setting [26].

Most recently, the European Society for Medical Oncology announced the results of a phase II multicenter study comparing cabozantinib and sunitinib in the first-line setting. One hundred fifty-seven patients were randomized to receive either agent, and the cabozantinib treatment group showed a 31% reduction in the median rate of progression or death compared to those treated with sunitinib [27]. Although the results are promising, more expansive studies will be needed including phase III studies to solidify the role of cabozantinib in patients with untreated mRCC.

### Monoclonal antibody targeting VEGF

Bevacizumab is a humanized recombinant monoclonal antibody against VEGF-A [28]. It was originally evaluated in a randomized phase II trial showing increased time to disease progression in patients who failed high-dose IL-2 [29]. Such promising data culminated in the AVOREN trial, a phase III, randomized, double-blinded study of 641 patients treated with bevacizumab plus IFN-α or placebo with IFN-α. Study results include bevacizumab benefit with mPFS (10.2 vs. 5.4 months), RR (30.6 vs. 12.4%), and a trend towards improved survival [30, 31]. A similar trial studying the viability of bevacizumab in the first-line setting (the CALBG trial) randomized patients to receive bevacizumab with IFN-α or IFN-α alone. In concordance with the AVOREN trial, the CALBG study showed that the patients in the bevacizumab arm had a greater mPFS and RR. These data propelled bevacizumab as a viable first-line agent alongside sunitinib and pazopanib therapies [32]. Bevacizumab has been studied in combination with sunitinib, sorafenib, or temsirolimus, and all encountered significant dose-limiting toxicities [30–33]. Currently, bevacizumab in combination with interferon has a category 1 NCCN recommendation for mRCC.

### mTOR inhibitors

A serine/threonine kinase and member of the PI3K family, mammalian target of rapamycin (mTOR), is implicated in the activation of a number of growth factors and signaling cascades, therefore having implications in tumorigenesis and angiogenesis [33–35]. Interestingly, the role of mTOR signaling in solid cancers was primarily investigated in the RCC model with the ultimate development of targeted agents for systemic therapy in mRCC patients [36, 37]. Over the past few years, experts have supported the growing body of evidence that there is a greater efficacy of VEGFR inhibitors compared to mTOR inhibitors in patients with mRCC [38]. Notwithstanding, temsirolimus and everolimus have had principal roles in mRCC in both the first-line and refractory setting.

Temsirolimus is the only mTOR inhibitor approved for the first-line treatment of mRCC with poor prognosis. In the phase III Global ARCC trial, temsirolimus was studied for first-line use in 626 previously untreated patients with mRCC with poor prognosis features based on MSKCC prognostic model. Participants were considered poor prognosis if three of the following six criteria were met: LDH ≥ 1.5 × ULN, Ca^++^ of ≥10 mg/dL, diagnosis to treatment initiation of <1 year, KPS 60–70%, ≥2 metastatic sites [39]. Of note, this study was indiscriminant of histological subtype and included both ccRCC and nccRCC subjects. ARCC compared IFN-α or a combination of temsirolimus and IFN-α. Temsirolimus monotherapy demonstrated a superior OS of 10.9 months compared to either IFN-α (7.3 months) or combination therapy (8.4 months). ARCC also reported temsirolimus to have a superior mPFS of 3.8 months over IFN-α (1.9 months) [40]. Temsirolimus is currently recommended by the NCCN for first-line use in patients with mRCC with features of poor prognosis.

While temsirolimus has not been compared directly with either sunitinib or pazopanib in the first-line setting for patients with mRCC, the issue of optimal sequencing between VEGF TKIs and mTOR inhibitors was addressed in the RECORD-3 trial using a different mTOR inhibitor, everolimus. RECORD-3 was a phase II study comparing the mPFS of treatment-naïve mRCC patients treated with sequential first-line everolimus and second-line sunitinib versus first-line sunitinib and second-line everolimus. The use of everolimus followed by sunitinib failed to demonstrate non-inferiority with regard to mPFS. To this end, study outcomes did not support the use of everolimus in first-line setting [41].

## Paradigm shift in the treatment of mRCC after anti-angiogenesis therapy

The primary challenge of mRCC is that complete response to treatment with a single agent is rare. Disease progression is expected and tumor resistance is an inevitable reality. mRCC remains incurable in most instances, and mechanisms of tumor-cell resistance to conventional radiotherapy and chemotherapy have been an active area of research. Proposed mechanisms include overexpression of multidrug resistance gene *MDR-1*, cell survival gene *clusterin*, PKC-ζ, L2 cell adhesion molecule L1-CAM, P-glycoprotein, various DNA repair proteins, the antiapoptotic gene *bcl-2*, glutathione S-transferase, decreased expression of DNA topoisomerase, loss of HIF-1α regulation, accumulation of HIF-2α, and suppression of p53 [40, 42]. Targeting these resistance mechanisms is an area of ongoing studies and may play a role in future approaches to the treatment of advanced RCC.

Prior to the approval of everolimus by FDA in 2009 for the second-line use in mRCC, there was no established treatment option for patients who progressed on first-line VEGF-directed therapy. Significant advancement has been made since, and the second-line treatment options now include TKIs—axitinib, cabozantinib, and lenvatinib; an anti-PD1 monoclonal antibody, nivolumab; and an mTOR inhibitor, everolimus. Sequential treatments have emerged as a viable approach to controlling drug resistance and overcoming resistance mechanisms [43], while the optimal sequence of treatment remains to be defined as few studies to date have directly compared drug efficacy [44].

### Everolimus

The oral agent mTOR inhibitor, everolimus, was the first drug to be approved for second-line use in mRCC after progression on first-line VEGF TKI treatment. In the RECORD-1 trial, everolimus was compared to placebo in 410 mRCC patients whom had been previously treated for at least 6 months with sunitinib, sorafenib, or both [45]. The study’s primary endpoint was mPFS, and everolimus performed superiorly to placebo with a significant mPFS difference of 4.0 to 1.9 months, respectively. A follow-up trial, RECORD-4, prospectively followed mRCC patients on everolimus after the progression of disease on either sunitinib, additional VEGF TKIs, or cytokine therapy [46] and confirmed the mPFS benefit of everolimus. The role of everolimus has been investigated beyond the second-line setting and investigators proposed that mTOR inhibition may have a role in untreated nccRCC. The ASPEN trial was a multicenter, open-label, randomized phase II trial designed to determine the mPFS of sunitinib and everolimus in a subset of patients with histologically proven nccRCC. Results favored sunitinib over everolimus (8.3 vs. 5.6 months) and reaffirmed the role of everolimus solely as a second-line agent, irrespective of histological subtype [47].

### Sorafenib and axitinib

The role of second-line sorafenib has been investigated in the INTORSECT trial, which enrolled patients whom had progressed after sunitinib therapy and randomized them to receive either temsirolimus or sorafenib with a primary endpoint of mPFS and secondary endpoints of safety, ORR and OS. The study concluded there was no difference in mPFS between the two agents; however, sorafenib demonstrated a superior OS of 16.6 months over temsirolimus, 12.3 months. Both agents presented with an acceptable safety profile, and the adverse effects were consistent with the known toxicities of the drugs [48].

Axitinib is an oral, potent, small-molecule TKI that selectively inhibits VEGFR-1, VEGFR-2, and VEGFR-3 [49]. The role of axitinib in the second-line treatment of mRCC has been investigated in the phase III AXIS trial, which randomized 723 patients with mRCC whom had disease progression after first-line systemic therapy [41]. First-line therapy included sunitinib, cytokine therapy, bevacizumab plus IFN-α, or temsirolimus. The trial met its primary endpoint, mPFS, and axitinib was shown to offer a superior mPFS of 6.7 months compared to sorafenib, 4.7 months. There was, however, no difference in the OS between the axitinib-treated group and sorafenib-treated group [50]. Common axitinib toxicities observed in the study included diarrhea (55%), hypertension (40%), and fatigue (39%).

### Cabozantinib

The second-line agents everolimus and axitinib had become the standard of care in refractory disease, but the mPFS was only extended by a mere 3 to 5 months after disease advancement on first-line therapy [41, 48]. Within the past year, two novel VEGFR TKIs, cabozantinib and lenvatinib, have gained FDA approval for use in advanced RCC.

Cabozantinib is an oral, small-molecule TKI-targeting VEGFR that was originally approved for metastatic medullary thyroid cancer. In addition to VEGFR, cabozantinib targets receptor tyrosine kinases implicated in and relevant to mRCC; RET, KIT, AXL, and FLT3 [51]. A landmark study by Zhou et al. provided evidence that MET and AXL are upregulated in chronic sunitinib use and play a role in RCC tumor resistance to TKIs [52]. This data is in concordance with previous studies which suggest poor prognosis when MET/AXL are highly expressed by RCC tumor cells [53]. Cabozantinib was studied in the METEOR trial, which was a randomized, open-label, phase III trial comparing cabozantinib with everolimus in 658 patients with mRCC whom had advanced after TKI therapy. The study’s primary endpoint was mPFS and secondary endpoints were OS and ORR. The rate of disease progression with cabozantinib was 42% lower than with everolimus. The METEOR trial met its primary endpoint, and cabozantinib demonstrated a superior mPFS of 7.4 months compared to 3.8 months with everolimus. An OS advantage was observed with cabozantinib (21.4 compared to 16.5 months), and the ORR significantly favored cabozantinib to everolimus, 21 to 5% (*p* < 0.001) [38]. Cabozantinib was observed to have a similar safety profile to drugs in its own class (Table 1). The incidence of grade 3 and 4 adverse effects was 68% with cabozantinib. The most common events were hypertension (15%), diarrhea (11%), and fatigue (9%). Dose reductions occurred in 60% of the study patients stratified to cabozantinib treatment. A grade 5 adverse event occurred in one patient [54].

**Table 1:** Common adverse effects of novel agents approved for mRCC

Adverse effect	Cabozantinib *N* = 331		Nivolumab *N* = 406		Lenvatinib *N* = 52	
	Any grade (%)	Grade 3/4 (%)	Any grade (%)	Grade 3/4 (%)	Any grade (%)	Grade 3/4 (%)
Diarrhea	85	11	13	1	72	12
Fatigue	65	9	35	2	50	8
Arthralgia/myalgia	11	<1	(11–21)	(0)	25	0
Decreased appetite	48	2	12	<1	58	4
Vomiting	34	2	(15–17)	(0)	39	4
Nausea	54	4	14	<1	62	8
Stomatitis	24	2	2	0	25	2
Hypertension	52	15	Not defined	Not defined	48	17
Peripheral edema	9	0	4	0	15	0
Cough	18	<1	9	0	17	2
Abdominal pain	20	4	(11–13)	(0)	31	4
Dyspnea	22	3	7	1	21	2
Decreased weight	33	2	Not defined	Not defined	48	6
Palmer-plantar erthrodysesthesia	50	8	Not defined	Not defined	15	0
Constipation	25	<1	(9–23)	(0)	37	0
Pruritus	8	0	14	0	6	0
Rash	15	<1	10	<1	17	0
	Choueiri et al. 2015 [54]		Motzer et al. 2015 [72] CheckMate 025 Trial		Motzer et al. 2015 [60, 69, 72]	

Common adverse reactions observed in patients with mRCC treated with novel therapies. The incidences reported have been extracted from the clinical trials leading to each agents FDA approval, respectively. Reported incidence in parentheses were extracted from general drug data, not specific to mRCC and not from the indicated study

The METEOR trial was published in November of 2016, and by April 2016, the FDA had approved cabozantinib as second-line treatment for advanced mRCC after anti-angiogenesis therapy. As to the question of where cabozantinib fits in the sequential treatment paradigm, its superiority to everolimus leaves axitinib as a possible comparator for a future study. Similar to the METEOR trial, the AXIS trial investigated disease refractory to sunitinib. Subgroup and post hoc analyses of the AXIS trial revealed that the patients whom had been treated with sunitinib and axitinib sequentially had a mPFS of 4.8 months and an ORR of 11% [41, 55]. Considering the 9.1-month mPFS and ORR of 22% observed in this study, cabozantinib could be a marked advancement in the treatment of mRCC. Moreover, the success of cabozantinib serves as a proof-of-principle that the targets (MET and AXL) that were not affected by previous drugs have an in vivo role in mRCC disease.

### Lenvatinib in combination with everolimus

One month after the FDA announcement of approval of cabozantinib for mRCC, lenvatinib was approved for the treatment of patients with advanced mRCC in combination with everolimus following disease resistance to TKIs. Lenvatinib is an oral, multi-target TKI of VEGFR1-3, FGFR1-4, PDGFRα, RET, and KIT [56]. First established as a therapy for differentiated thyroid cancer, lenvatinib has had a favorable antitumor profile with acceptable toxicities in a multitude of solid tumors in both phase I and II trials [57, 58]. The mechanism by which tumors develop VEGF resistance and develop compensatory angiogenesis pathways provides the rationale for studies on drugs with multiple targets [59]. Prior in vivo studies using mouse xenografts of human RCC showed a reduction in tumor volume with a lenvatinib and everolimus combination [60]. Further, in vitro binding studies reveal a highly specific binding site to the receptor kinase domain, suggesting possible limited toxic effects [61]. To this end, lenvatinib had been identified as a candidate for clinical studies in patients with advanced mRCC refractory to first-line agents.

The role of lenvatinib in the treatment of mRCC has been studied in a randomized, phase II, open-label, multicenter trial, which enrolled 153 patients with mRCC that progressed on first-line VEGF-directed therapy [62]. Patients were stratified in a 1:1:1 ratio and received lenvatinib, everolimus, or combination therapy with a primary endpoint of mPFS. Lenvatinib plus everolimus significantly prolonged mPFS compared to everolimus, 14.6 to 5.5 months, but not to single-agent lenvatinib, 7.4 months. Moreover, OS was increased in the group receiving the dual-therapy compared to everolimus alone, although not statistically significant. Single-agent lenvatinib significantly prolonged mPFS compared to everolimus as well. However, the size of the benefit of the combination therapy as compared to the benefit of single-agent lenvatinib suggests that efficacy was most robust with the combination therapy [63]. The design of this three-armed study not only provides objective clinical data but also presents an emerging concept in mRCC therapy that combination drugs targeting multiple pathways (in this case VEGF and mTOR) could simultaneously inhibit two critical independent pathways synergistically and can potentially prevent resistance to single-agent therapy [62].

The toxicity profile of the combination therapy was consistent with the known toxicities of each individual agent (Table 1). Expectedly, the combination therapy exhibited more frequent adverse events than either single therapy. These most common grade 3 and 4 treatment-emergent adverse events from the dual-therapy patient group include constipation (37%), diarrhea (20%), fatigue (14%), and hypertension (14%). The increased likelihood of toxicity is an appreciable concern and should be considered by clinicians when deciding upon second-line therapy.

### Immunotherapy with PD-1 and PD-L1 inhibitors

mRCC is highly immunogenic. Neoplastic cells evade immune cell surveillance allowing for uninhibited and unregulated cell growth. The novel principle underlying immunotherapy entails the activation of the endogenous immune system to target cancer at the cellular level and enable checkpoint inhibition [64]. Immunotherapy agents at work in mRCC have a role in two-principle immune signaling mechanisms: (1) cytotoxic T-lymphocyte-associated antigen 4 (CTLA-4) and (2) programmed death receptor-1 (PD-1). These two receptors are negative immune regulators imperative to preventing autoimmunity and are endogenous signals for suppression of lymphocytes and natural killer (NK) cells. Although the ability of cancer cells to suppress the immune system is maladaptive, researchers have been able to use this facet of cancer biology as a target for drug development [65].

The targeted therapies reviewed above and immunotherapies have vastly different mechanisms of action; however, both treatment modalities show durable responses in mRCC tumor regression. Interestingly, studies have shown that targeted therapies are able to dramatically enhance immune cell function as well. Together, this creates a paradigm whereby immunotherapy and targeted therapy can be employed concomitantly with a synergistic effect [66]. Herein, we describe the immunotherapies approved by the FDA for mRCC including the newly approved agent nivolumab and highlight the promising data that led to nivolumab’s approval.

PD-1 is a cell surface glycoprotein expressed on T-lymphocytes, B-lymphocytes, macrophages, and NK cells. Ligand binding of the PD-1 receptor inhibits the effector phase of T cell activation and thereby serves as a potent immune checkpoint receptor [6]. The pathogenesis of mRCC cells and their immunomodulatory effect stems from the interface between malignant cells and the PD-1 receptor. The natural ligand to PD-1 is the PD-1 ligand (PD-L1), which is expressed on antigen-presenting cells. Importantly, populations of mRCC cells have also been shown to express PD-L1, and this mimicry can result in not only unregulated tumor cell growth but also apoptosis of antigen-specific T cells [11, 67]. This interplay between malignant cells and immune cells creates a tumor microenvironment with an active yet functionally impaired immune system. Studies have gone on to show that the degree of PD-L1 expression on mRCC cells directly correlates with aggressive pathologic features including advanced TNM staging, tumor size, higher nuclear grade, coagulative necrosis, increased disease progression, cancer-specific death, and overall mortality [68].

Nivolumab is a fully humanized immunoglobulin G4 PD-1 immune checkpoint inhibitor antibody that selectively blocks the receptor activation of PD-L1 and PD-L2. Ultimately, nivolumab enhances T cell function which results in antitumor activity [69]. Nivolumab has changed the landscape for multiple solid and liquid tumors and currently holds FDA approval for the treatment of melanoma, squamous non-small cell lung cancer, and classical Hodgkin’s lymphoma as well as mRCC. The CheckMate016 phase I trial included patients with mRCC and was first presented at ASCO 2015. This study demonstrated that a combination of nivolumab and ipilimumab exhibited a durable antitumor effect with a manageable safety profile [70]. A recently reported 5-year follow-up investigation of the phase I participants revealed a 34% 5-year survival rate for mRCC patients whom failed prior anti-angiogenesis therapy and then placed on maintenance nivolumab [71].

Building on these promising results, investigators recruited 168 patients with histological confirmation of mRCC for a phase II study. The patient had received prior treatment with either a VEGF TKI or VEGF monoclonal antibody and suffered from progression of disease. Patients were stratified to receive varying doses of nivolumab as a single-agent therapy. The study successfully demonstrated nivolumab to prolong mPFS (2.7–4.7 months), ORR (20–22%), and mOS (18.2–25.5). Together, the results of the study suggested promising antitumor activity while exhibiting minimal systemic toxicities [69].

The abovementioned results were encouraging as a proof-of-principle validating immunotherapy as a treatment option for mRCC. CheckMate 025 trial was the first phase III randomized controlled trial examining nivolumab in advanced RCC. Nivolumab was compared to everolimus in disease refractory to VEGFR targeted therapy. Eight hundred twenty-one patients were stratified to receive either nivolumab or everolimus with a primary endpoint of OS. Secondary endpoints included ORR and safety. The results from this study were remarkable and ultimately led to FDA approval of the drug. mOS was 25.0 versus 19.6 months for nivolumab and everolimus, respectively, and met the predetermined criteria for significance. The hazard ratio (HR) for nivolumab met superiority over everolimus (HR 0.73, *p* < 0.0148). Further significant findings indicating nivolumab superiority included an ORR of 25 versus 5% and an OR of 5.98. There was no difference in mPFS between the two agents. Moreover, the safety and tolerability profiles favored nivolumab over everolimus (Table 1). Nineteen percent of the nivolumab-treated patients reported grade 3 or 4 treatment-related adverse effects compared to 37% of the patients whom had received everolimus. Of note, the durable responses seen by nivolumab were irrespective of MSKCC prognostic score, number of previous anti-angiogenic therapies, and PD-L1 expression [72]. Data extrapolated from the CheckMate 025 trial analyzed the role of nivolumab treatment after progression, an emerging concept in clinical oncology [73]. With regard to the patients in the study treated with nivolumab, 171 of the enrolled patients were treated beyond progression of disease. The patients treated beyond progression experienced a mOS of 28.1 months compared to 15.0 months for the group not treated after progression (*p* < 0.001) [74]. These data are suggestive that the immune response of nivolumab may be delayed, and further studies are necessary to fully elucidate the timing of the clinical effect of the drug.

A 2016 study building off the results from the CheckMate 025 trial compared health-related quality of life (HRQoL) for patients in the treatment groups of this trial. Nivolumab was associated with an improved HRQoL compared to everolimus in this study population [75]. Although there was no significant difference in the mPFS observed, ad hoc sensitivity analysis of mPFS in the patients whom had not progressed or died within six months of treatment revealed a delay in progression on nivolumab that achieved statistical significance. The landmark CheckMate 025 trial and subsequent studies have resulted in labeling nivolumab as the leading monotherapy for second-line therapy for those who fail VEGFR-targeting therapies [76].

More recent studies have shown that the immunomodulatory effect of nivolumab in the mRCC tumor microenvironment is expansive [77]. In an elegantly designed study, baseline and on-treatment biopsies were obtained from mRCC patients receiving nivolumab therapy (both treatment-naïve and refractory disease patients). Immunohistochemical analysis of these biopsies demonstrated an increased lymphocytic presence in the nivolumab-treated group, reversal of T cell exhaustion within the tumor microenvironment, upregulation of genes that are hallmarks of the Th1 inflammatory response, and increased tumor trafficking or infiltration of T cells. The investigators also report an increase in expression of genes linked to NK cells, suggesting that the immunomodulatory effect of nivolumab may be augmented with NK cell-directed therapies in the future [78].

## Treatment selection after anti-angiogenesis therapy

The optimal sequencing of mRCC treatment beyond first-line VEGF TKIs remains controversial, as there are no head-to-head comparisons between currently approved drugs. Everolimus was the first drug approved for second-line treatment of mRCC based on mPFS benefit over placebo; however, objective tumor response was low, and no overall survival benefit had been demonstrated [48]. Nivolumab is arguably the most promising agent with a unique immune mechanism of action and showing an OS benefit over everolimus in the second-line treatment of mRCC. In contrast to previously held belief, there was no significant difference in the median time to progression for nivolumab versus everolimus [72]. Lenvatinib in combination with everolimus, in a phase II trial, demonstrated to offer mPFS benefit over single-agent everolimus, but not over single-agent lenvatinib. Further studies are needed to define the role of lenvatinib, and the cost of combination therapy may be an issue to consider in clinical practice. The OS benefit of both these agents over everolimus is remarkable and solidifies both agents as marked improvements over the therapies previously available for use in clinical practice.

Among TKIs currently approved for second-line use, only axitinib and sorafenib have been compared in a head-to-head fashion. Axitinib is more potent and selective than sorafenib. In the AXIS trial, it demonstrated mPFS benefit over sorafenib, but no overall survival benefit [55]. Cabozantinib has the theoretical benefit of additional inhibition of MET and AXL, which are believed to play a role in the resistance of mRCC to VEGF-directed therapy [57]. Cabozantinib remains the only agent with OS, mPFS, and ORR benefit over everolimus [38]. At the moment, cabozantinib is the preferred TKI in our opinion; however, argument can be made for reserving its use for progression of disease, as cabozantinib was shown to be effective in METEOR trial for patients previously treated with more than one line of VEGF TKIs.

The approach to the decision of drug sequence requires consideration of the drug’s side-effect profile and patient preference. Nivolumab is a biweekly intravenous infusion while cabozantinib is an oral medication that is more convenient but subject to patient compliance. The most common side effect of nivolumab appears to be fatigue, while cabozantinib causes more diarrhea and hand-foot syndrome.

There is no level 1 data to guide the choice between nivolumab and cabozantinib, and the argument can be made in favor of either. A recent study compared the efficacy of nivolumab and cabozantinib through the analysis of data from multiple trials. By extracting survival data from original study data and analyzing the hazard ratio over time, the authors found that the hazard ratio for OS favors cabozantinib in the first few months but then nivolumab afterwards [79]. A conclusion may be drawn that patients with poor prognosis benefit more from cabozantinib while patients with a better prognosis will benefit more from nivolumab. This conclusion is supplemented by the latest update from the METEOR trial, which provides direct evidence supporting the use of cabozantinib in high-risk patients [38]. On the other hand, data from the subgroup analysis of CheckMate 025 trial suggests nivolumab is of benefit across subgroups including high-risk patients.

We have learned from the INTORSECT trial that sorafenib offers superior OS over temsirolimus in the second-line setting and that continued VEGF-directed therapy beyond first-line sunitinib is more efficacious than mTOR inhibitor [43]. In the following years, the large multicenter AXIS, CheckMate, METEOR, and INTORSECT trials introduced a multitude of new promising agents (Table 2). Though these studies have established a role for novel therapies, there is a degree of disconnect between the individual studies and the overall treatment landscape for mRCC.

**Table 2:** Major clinical trials for the treatment of mRCC after anti-angiogenesis therapy

Study	Everolimus	Axitinib	Sorafenib	Cabozantinib	Nivolumab	Lenvatinib/everolimus
	Record-1	Axis	Intorsect	Meteor	Checkmate 025	NCT01136733 (phase II)
Control	Placebo	Sorafenib	Temsirolimus	Everolimus	Everolimus	Everolimus
Population	Metastatic RCC progressive on sunitinib, sorafenib, or both	Metastatic RCC progressive on either sunitinib, bevacizumab/IFNα, temsirolimus, or cytokine-based regimen (only 1 line of treatment allowed)	Metastatic RCC progressive on sunitinib	Metastatic RCC progressive on at least one VEGFR-targeting inhibitor but no limit on the number of lines of prior treatment	Metastatic RCC progressive on one or two anti-angiogenic therapy	Metastatic RCC progressive on one line of VEGF-directed therapy
Crossover allowed?	Yes	No	Not specified	No	Not specified	Not specified
1° endpoint	PFS *4.9 months (everolimus) vs. 1.9 months (placebo)*	PFS *8.3 months (axitinib) vs. 5.7 months (everolimus)*	PFS 3.9 months (sorafenib) vs. 4.3 months (temsirolimus)	PFS *7.4 months (cabozantinib) vs. 3.8 months (everolimus)*	OS *25 months (nivolumab) vs. 19.6 months (everolimus)*	PFS *14.6 months (lenvatinib/everolimus) vs. 5.5 months (everolimus)*
2° endpoints						
OS	14.8 months (everolimus) vs. 14.4 months (placebo)	20.1 months (axitinib) vs. 19.2 months (everolimus)	*16.6 months (sorafenib) vs. 12.3 months (temsirolimus)*	*21.4 months (cabozantinib) vs. 16.5 months (everolimus)*	See above	25.5 months (lenvatinib/everolimus) vs. 17.5 months (everolimus)
ORR	1.5% (everolimus) vs. 0% (placebo)	19% (axitinib) vs. 9% (everolimus)	8% in both arms	21% (cabozantinib) vs. 5% (everolimus)	25% (nivolumab) vs. 5% (everolimus)	43% (lenvatinib/everolimus) vs. 6% (everolimus)
PFS	See above	See above	See above	See above	4.6 months (nivolumab) vs. 4.4 months (everolimus)	See above
Median time to response	Not provided	Not provided	Not provided	Not provided	3.5 months (nivolumab) vs. 3.7 months (everolimus)	Not provided
Duration of response	Not provided	11 months (axitinib) vs. 10.6 months (everolimus)	Not provided	Not provided	12 months for both arms	13 months (lenvatinib/everolimus) vs. 8.5 months (everolimus)
Discontinuation rate for toxicity	10% (everolimus) vs. 4% (placebo)	4% (axitinib) vs. 8% (everolimus)	Not provided	9% (cabozantinib) vs. 10% (everolimus)	8% (nivolumab) vs. 13% (everolimus)	Not provided
Grade 3–4 toxicity	Everolimus: stomatitis (3%), infections (3%), pneumonitis (3%)	Axitinib: hypertension (16%), diarrhea (11%), fatigue (11%)	Sorafenib: palmar-plantar erythrodysesthesia (15%), rash (3%), and fatigue (7%)	Cabozantinib: hypertension (15%), diarrhea (11%), fatigue (9%)	Nivolumab: fatigue (2%)	Lenvatinib/everolimus: diarrhea (20%)

*OS* overall survival, *PFS* progression-free survival, *ORR* overall response rate

Italics indicate statistical significance in data findings

**Fig. 1:**
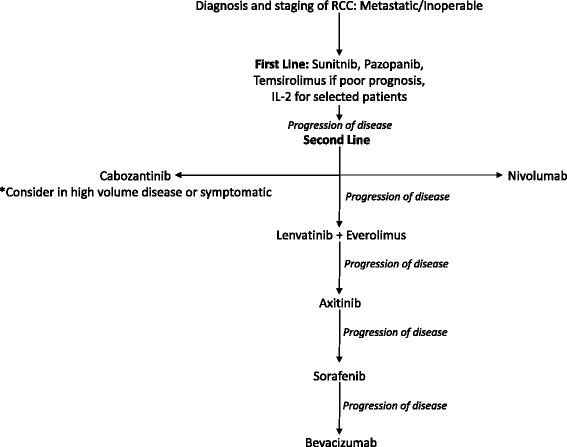
Suggested approach to treatment after anti-angiogenesis therapy. Suggested algorithm for treatment options for mRCC. Sunitnib and pazopanib are recommended in the first-line setting, with the exception of selected patients who may benefit from temsirolimus or IL-2. Upon disease progression, second-line agents can be chosen at the discretion of the clinician. The algorithm provided is based on current clinical data and practice guidelines

In our opinion, we recommend choosing either cabozantinib or nivolumab for patients with mRCC who progressed on first-line VEGFR-directed therapy, with cabozantinib preference for patients with high volume disease or symptoms due to its PFS benefit (Fig. 1). Both are acceptable options despite no current level 1 data to support the use of one agent over the other. Lenvatinib with everolimus, too, is a viable option in this setting with its appeal being dual therapy and multiple targets. Drug adverse effects and patient preference consideration should play the deciding role. In practice, we suggest substituting to the alternative agent upon progression of disease before considering everolimus, as both are superior to everolimus in OS. Given the data from the INTORSECT trial, we would consider the use of alternative VEGFR TKIs, e.g., axitinib and sorafenib, prior to the use of everolimus. This recommendation is offered with the caveat that neither has been directly compared to everolimus. Lastly, clinical trial participation is always encouraged during any stage of treatment. The CheckMate214 trial is an ongoing phase II trial comparing nivolumab and ipilimumab versus sunitinib in the first-line setting [80] (Table 3). Such trials assessing immunotherapies are most promising to fully elucidate the survival benefit immunotherapies will have compared to the TKI and monoclonal antibody class agents.

**Table 3:** Active clinical trials investigating future immunotherapies in mRCC

Trial	Phase	Estimated completion	Disease setting	Standard treatment	Experimental treatment
NCT01582672	III	April 2017	Advanced renal cell carcinoma	Sunitinib	Sunitinib + AGS-003
NCT02459067	II/III	December 2017	Refractory: Malignant Melanoma NSCLC Renal cell cancer	None	ImmuniCell®
NCT02917772	II	April 2018	Advanced renal cell carcinoma	None	Nivolumab + ipilimumab
NCT02718066	Ib/II	September 2017	Refractory: Malignant Melanoma NSCLC Renal cell cancer	None	Nivolumab + HBI-8000
NCT02853331	III	December 2019	Metastatic ccRCC	Sunitinib	Pembrolizumab + axitinib
NCT02684006	III	June 2018	Metastatic ccRCC	Sunitinib	Avelumab + axitinib
NCT02231749	III	June 2019	Advanced renal cell carcinoma	Sunitinib	Nivolumab + ipilimumab
NCT02420821	III	July 2020	Advanced renal cell carcinoma	Sunitinib	Bevacizumab + atezolizumab
NCT02811861	III	October 2019	Metastatic ccRCC	Sunitinib	Lenvatinib + everolimus OR Lenvatinib + pembrolizumab

Active trials investigating the roles of various immunotherapies in advanced and metastatic RCC. All trial information obtained through publicly accessible *clinicaltrials.gov*. AGS-003 is an autologous dendritic cell immunotherapy. ImmuniCell® is an autologous γδ T-lymphocyte immunotherapy. HBI-8000 (Chidamide) is a novel oral histone deacetylase inhibitor and epigenetic modulator

## Conclusions

Long-term control of disease in the treatment of mRCC has been a challenge due to drug resistance. We have witnessed the armamentarium of treatment options for mRCC rapidly evolve, including the approval of the novel agents cabozantinib, nivolumab, and lenvatinib. mPFS and OS have been prolonged, toxicities reduced, and treatment options have been extended to the third- and fourth-line settings with the design of targeted and immunotherapies. Additionally, a number of active clinical trials are examining agents for future use in mRCC and will further expand the list of FDA-approved drugs (Table 3). However, the tremendous rate by which novel agents are being designed has created a level of complexity which clinicians must manage; treatment plans for patients have become highly variable with fewer studies assessing optimal sequence or combination of agents [64]. The question of sequencing is not unique to mRCC, as this dilemma is commonly faced when multiple therapeutic agents are developed over a short period of time for any disease process [44]. Thankfully, there are a number of randomized phase II and III trials currently ongoing that are examining both sequencing and combinatorial effects of already employed agents.

Future directions for the management of mRCC are not limited to studies investigating optimal sequence of therapies. There remains ambiguity as to the response of different histological subtypes of RCC to standard treatments [47]. There have been a number of studies in this area; however, the complexity of the various molecular mechanisms behind each subtype requires more expansive research efforts. Histological and pathological variants differ in disease biology, clinical behavior, prognosis, and response to systemic therapy [81]. Characterizing the molecular basis for each subtype will allow for greater precision for future clinical trial design with regard to targeted therapy and choice of agents. Active clinical trials are investigating possible synergism between concomitant targeted therapy and immunotherapy (Table 3). The potential of these studies, together with the advances in genomic medicine, provides a promising outlook for future care of patients with RCC.

In this review, we have highlighted the current landscape for mRCC treatment in the first-line and second-line settings. The anti-angiogenesis agents, sunitinib and pazaopanib, have remained the most utilized first-line agents [82]. After the use of these agents, axitinib and everolimus have been used extensively, but now, new agents including cabozantinib, nivolumab, and lenvatinib in combination with everolimus have provided a paradigm shift for the treatment of patients with prior anti-angiogenesis therapy. These novel therapies have gained traction for use in the second-line setting since their FDA approval. Questions remain as to what is the optimal selection of drugs in this setting. The combination of novel immunotherapies with targeted therapy has potential to dramatically improve the outcome for patients with mRCC.

## Abbreviations

ccRCC: Clear cell renal cell carcinoma
CTLA-4: Cytotoxic T-lymphocyte-associated antigen 4
HRQoL: Health-related quality of life
IFN-α: Interferon-alpha
IL-2: Interleukin-2
mOS: Median overall survival
mPFS: Median progression-free survival
mRCC: Metastatic renal cell carcinoma
mTOR: Mammalian target of rapamycin
NCCN: National Comprehensive Cancer Network
nccRCC: Non-clear cell renal cell carcinoma
NK: Natural killer
ORR: Objective response rate
OS: Overall survival
PD-1: Programmed death receptor-1
PD-1L: PD-1 ligand
PDGF: Platelet-derived growth factor
PFS: Progression-free survival
RCC: Renal cell carcinoma
TKI: Tyrosine kinase inhibitor
VEGF: Vascular endothelial growth factor
VEGFR: Vascular endothelial growth factor receptor
